# Supplementary material for: 4D imaging reveals mechanisms of clay-carbon protection and release
Source: Nat Commun. 2021 Jan 27;12:622. doi: 10.1038/s41467-020-20798-6 (PMC7840981; doi:10.1038/s41467-020-20798-6)
Supplement: Supplementary file 1 — Supplementary Information [file 41467_2020_20798_MOESM1_ESM.pdf]

Supplementary Information

4D imaging reveals mechanisms of clay-carbon protection and release

Judy Q. Yang[1], Xinning Zhang[2,3], Ian C. Bourg[3,4*] & Howard A. Stone[1*]

[1]Department of Mechanical and Aerospace Engineering, Princeton University, Princeton, NJ 08544, USA. [2]Department of Geosciences, Princeton University, Princeton, NJ 08544, USA. [3]Princeton Environmental Institute, Princeton University, Princeton, NJ 08544, USA. [4]Department of Civil and Environmental Engineering, Princeton University, Princeton, NJ 08544, USA. *email: bourg@princeton.edu and hastone@princeton.edu.

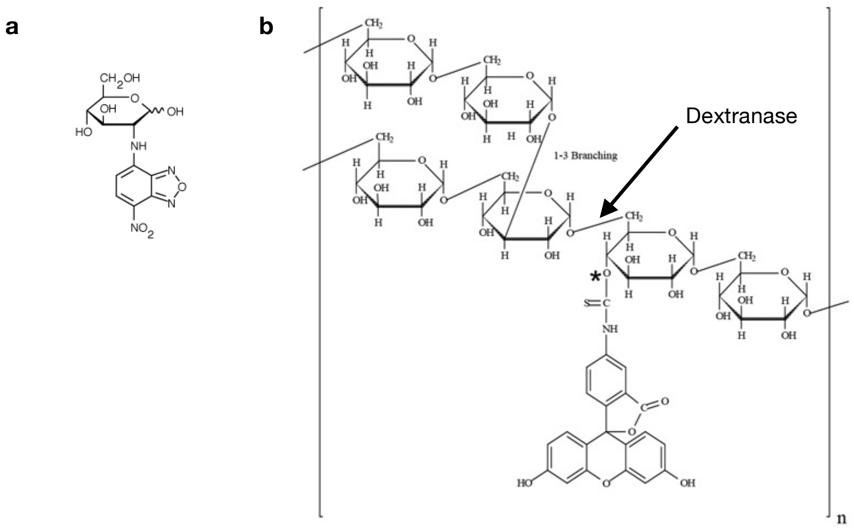

Supplementary Figure 1. Structures of 2-NBDG glucose (a) and FITC dextran (b). Images are from (a) Thermo Fisher and (b) Sigma Aldrich websites. Using Dynamic Light Scattering in the Princeton Biophysics Core Facility, we estimated that the hydrodynamic radii of 3-5 kDa dextran, 20 kDa dextran, and 70 kDa dextran are 2 nm, 5 nm, and 6 nm, respectively. The hydrodynamic radius of 2-NBDG glucose was too small to measure. The linkage that the enzyme dextranase breaks down is indicated by the black arrow. The hydrodynamic radius of the dextranase is 7 nm.

Supplementary Table 1. Molecular weights of the organic substances used in this study

**Name**	2-NBDG glucose	Fluorescein Isothiocyanate-Dextran (FITC dextran)	Tetramethylrhodamine isothiocyanate-Dextran
**Molecular weight**	342 Da	3-5 kDa, 70 kDa, 500 kDa, 2000 kDa	20 kDa

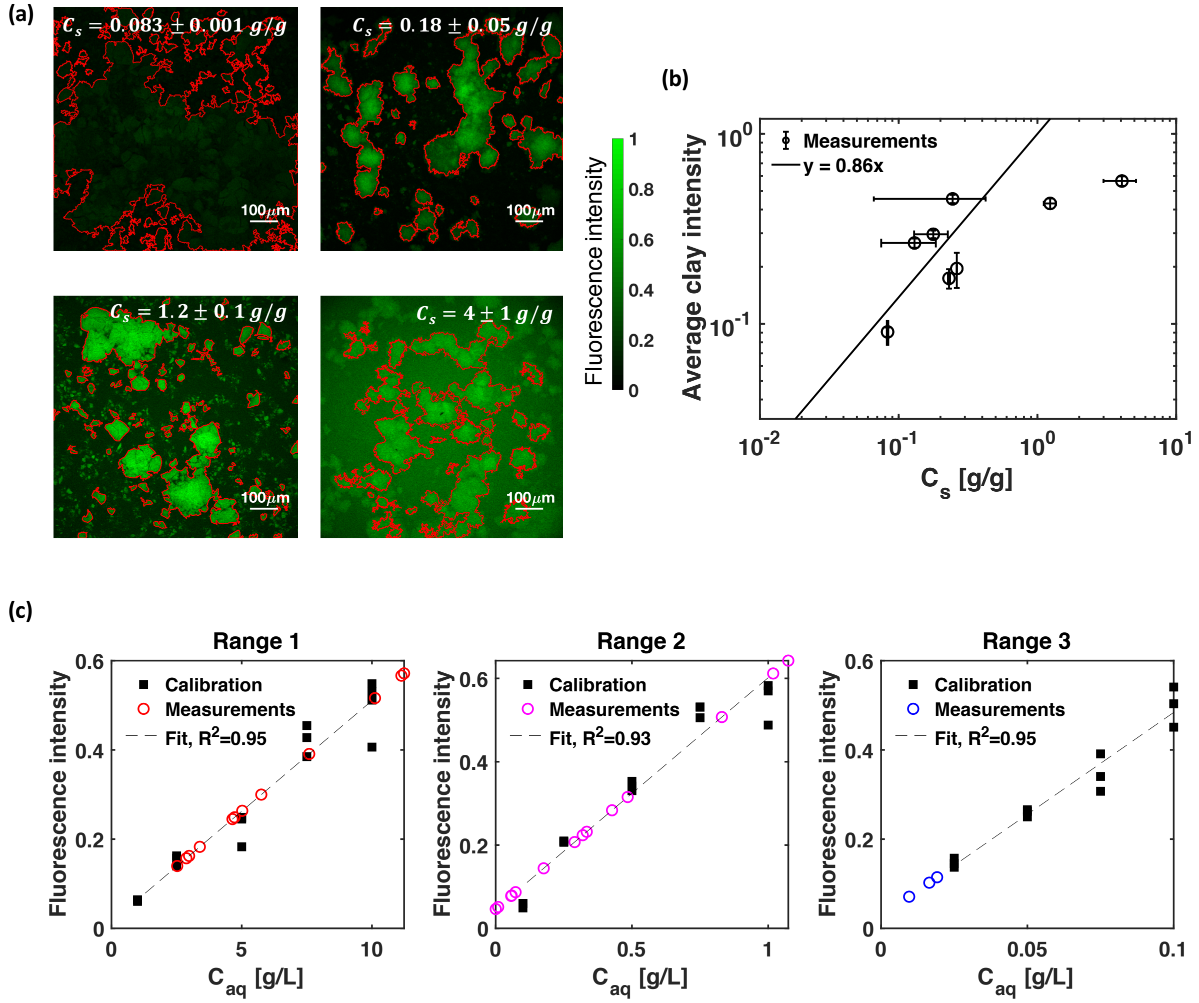

Supplementary Figure 2. Calibration of the average fluorescence intensity of clay relative to the calculated concentration of organic matter sorbed to clay. (a) Cross-sectional images of clay aggregates after soaking for three days in solutions with fluorescent organic matter (3-5 kDa dextran) of different concentrations. The clay areas, shown by the red contours, were identified as patches of pixels with fluorescence intensity larger than the average intensity of the whole image. (b) The average fluorescence intensity within the clay increases with the organic matter concentration in clay C_s in an approximately linear manner at $C_s < 1$ g/g, with correlation coefficient $R = 0.6$ (in log scale), and then reaches a plateau (fluorescence intensity ≈ 0.7). The black line represents the linear regression between the average clay intensity and C_s in log scale at $C_s < 1$ g/g. The linear to plateau relationship is consistent with the fact that fluorescence intensity generally increases with increasing concentration of the fluorescent labels before the intensity of the fluorescent image saturates[1]. The laser intensity used to excite the fluorescence was the same for all measurements. The error bars are the standard errors of multiple imaging measurements from the same sample (same batch experiment). C_s was calculated from the fluorescence intensity of the supernatant solution, C_{aq}, before and after adding clay (see Methods). (c) The concentration of organic matter in solution, C_{aq} (open circles), was interpolated from calibration curves of average fluorescence intensity versus concentration (dashed lines), which were derived by measuring the fluorescence intensity

for solutions with known dextran concentration (squares) in three different ranges. Three different laser intensities were used to resolve the fluorescence intensity in these three ranges. Note that the fluorescence intensity in the clay cannot be directly compared with the fluorescence intensity in the solution because clay intensifies the emitted fluorescence intensity.

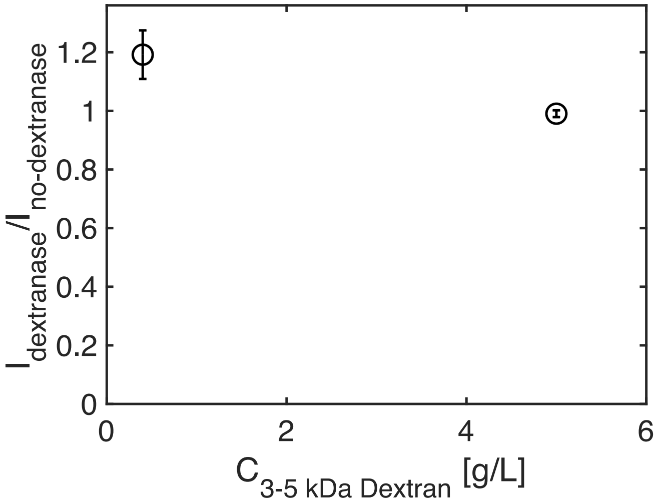

Supplementary Figure 3. Impact of dextranase on the average fluorescence intensity of fluorescent dextran. The fluorescence intensities of green FITC 3-5 kDa dextran in buffer solution with and without 2 g/L dextranse, i.e., $I_{dextranase}$ and $I_{no\text{-}dextranas}$, shows less than 20% difference, which is insignificant considering that the fluorescence intensity varies by one order of magnitude during the sorption and desorption experiment.

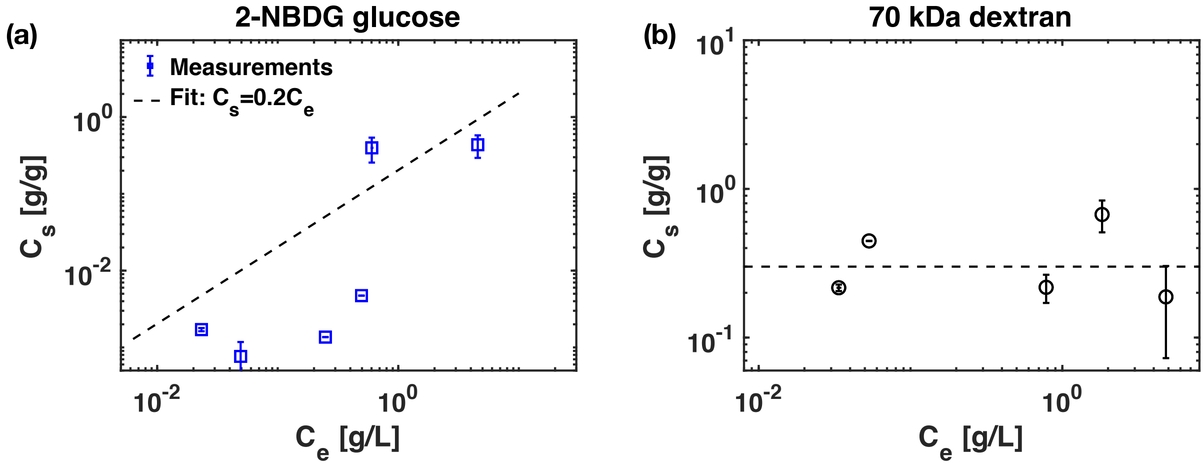

Supplementary Figure 4. Clay-carbon sorption isotherms of 2-NBDG glucose and 70 kDa dextran. (a) The concentration of glucose sorbed to clay, C_s, increased with increasing concentration of glucose in the solution, C_e. The error bars are standard errors of multiple measurements from one sample. The scatter of the data, especially at low carbon concentration, is likely because C_e and C_s were estimated from the fluorescence intensity of the supernatant (see Methods and Supplementary Fig. 2), which was difficult to measure accurately at low concentration when the fluorescent signal was comparable to the background noise. Despite the scatter, the C_s versus C_e data are roughly consistent with the expectation of a linear adsorption isotherm (exemplified by the dashed line with a slope of 1). (b) The concentration of 70 kDa

dextran sorbed to clay, C_s, was essentially invariant with C_e values ranging over two orders of magnitude. The plateau loading of 70 kDa dextran is essentially identical to that of 3-5 kDa dextran (Fig. 2(a)), $C_{s\text{-}plateau}$ = 0.2 ± 0.1 g/g, as expected if this plateau corresponds to the formation of a monolayer of high molecular-weight organic matter on the clay surface. Note that the upper range of C_e in this study was limited by the solubility of glucose and dextran in water, which is on the order of 10 g/L.

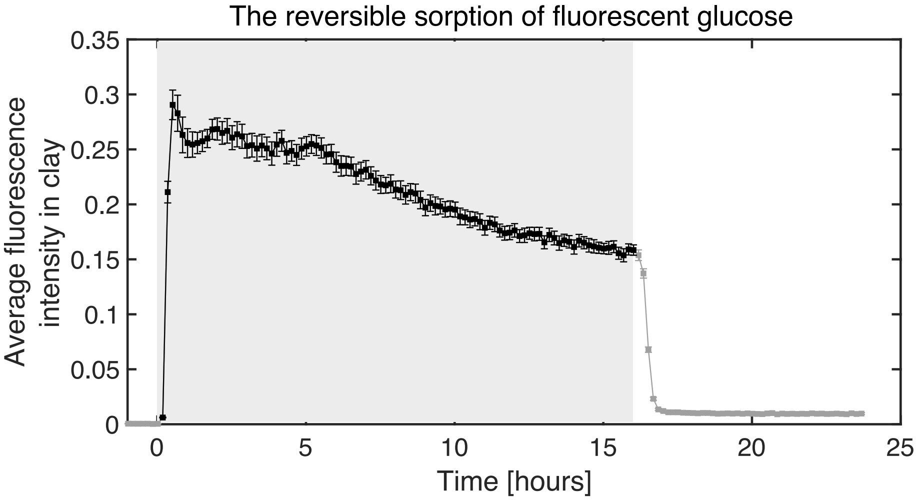

Supplementary Figure 5. Reversible sorption of glucose to clay was consistently observed in replicate sorption/desorption experiments. The symbol and the error bar represent the mean and the standard error of the average fluorescence intensity within 5 representative clay aggregates in one channel. The selected 5 clay aggregates were indicated by the red boxes in Supplementary Fig. 7 (b). The experiment is similar to the one shown in Fig. 1(e), except that the sorption period (the gray region) is about seven times longer than in Fig. 1. The decrease in fluorescence intensity of 2-NBDG glucose during the sorption period likely reflects photobleaching of the fluorescent molecules.

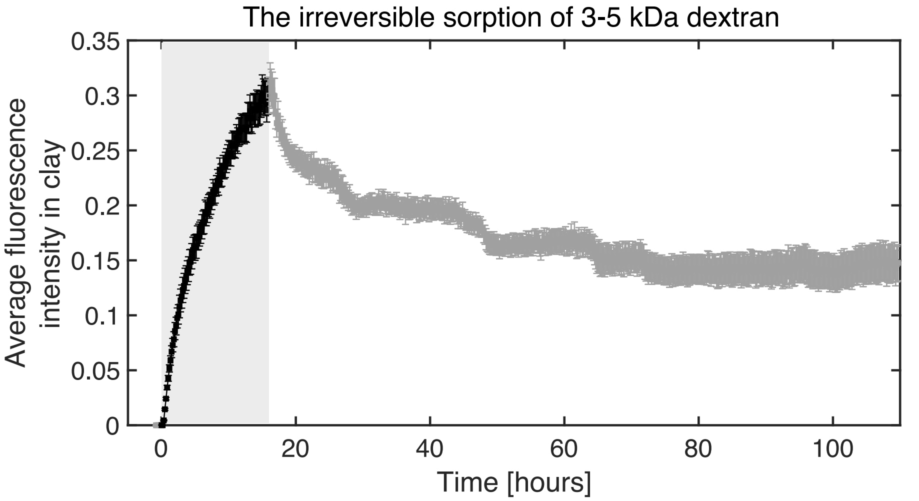

Supplementary Figure 6. Quasi-irreversible sorption of 3-5 kDa dextran to clay was consistently observed in replicate experiments. The symbol and the error bar represent the mean and the standard error

of the average fluorescence intensity within 5 representative clay aggregates in one channel. The selected 5 clay aggregates (or groups of clay aggregates) were indicated by the red boxes in Supplementary Fig. 7 (c). The experiment is similar to the one shown in Fig. 1(d), except that the fluorescence intensity was scanned every 10 minutes, less frequently than the 1-minute scanning interval used in Fig. 1(d). Because of the lower scanning frequency, the photobleaching of dextran was slower than in Fig. 1(d) during the desorption period. Note that compared with Fig. 1(d), the sorption of the dextran did not reach equilibrium at 16 hours, perhaps because the clay aggregates were more densely packed in this experiment (Supplementary Fig. 7(c)), suggesting that the kinetics of organic matter uptake depend on the microstructure of the clay aggregates. The type of sorption, i.e., quasi-irreversible sorption, was consistent with Fig. 1(d).

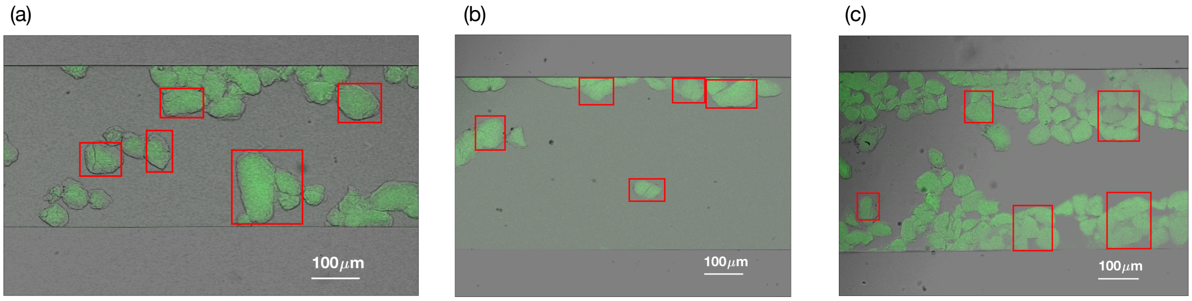

Supplementary Figure 7. Cross sectional images showing the arrangements of clay aggregates in microfluidic channels. The red boxes in (a), (b), and (c) show the 5 clay aggregates used to estimate the fluorescence intensity of carbon for the experiments shown in Fig. 1 (e), Supplementary Fig. 5, and Supplementary Fig. 6, respectively. The channel width is 300 μm for (a) and 400 μm for (b) and (c).

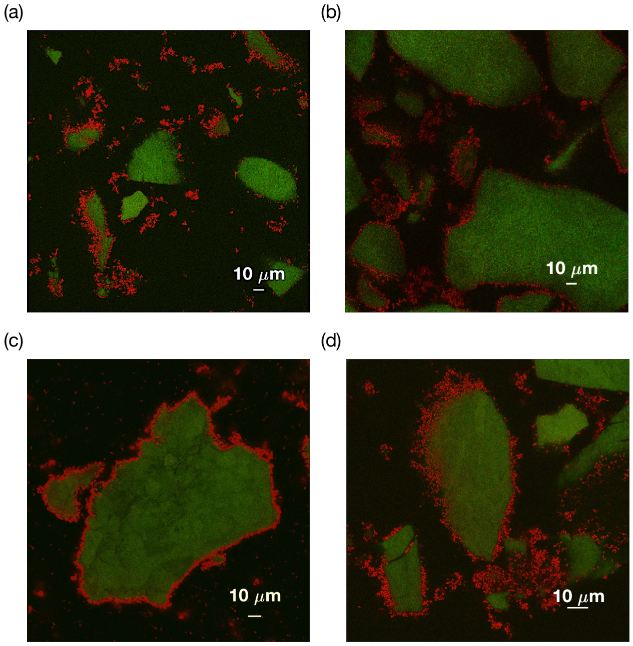

Supplementary Figure 8. Replicate bacteria-clay culture experiments consistently show that bacteria are excluded outside clay aggregates. The images were scanned with different resolutions, e.g. 0.48 μm, 0.48 μm, 0.24 μm, 0.03 μm for (a), (b), (c), and (d), respectively.

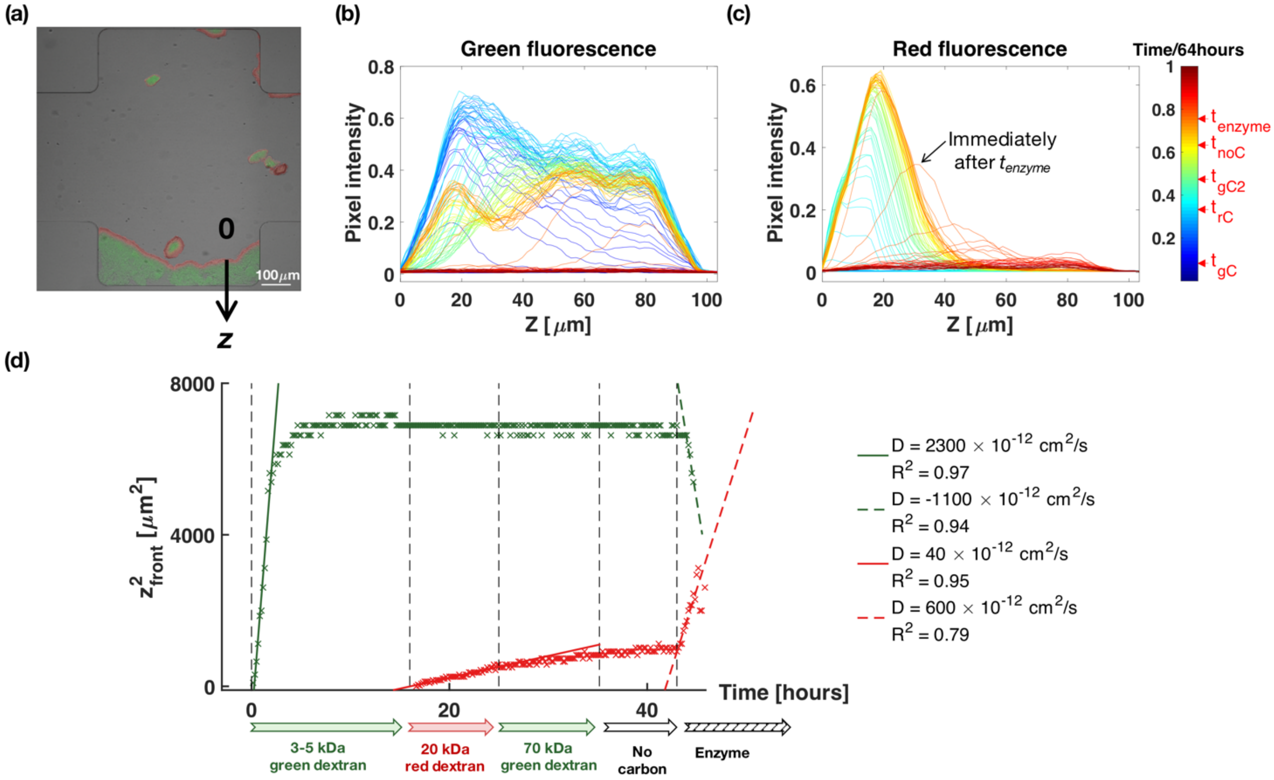

Supplementary Figure 9. Cross-sectional profiles of dextran fluorescence intensity in clay and the diffusivity of dextrans in clay. (a) Cross-sectional fluorescent image of a microfluidic channel containing clay micro-aggregates, the same experiment as in Fig. 3. The green and red fluorescence intensities along a transect, the z-axis in (a), were plotted over time in (b) and (c), respectively; $z = 0$ µm denotes the edge of the clay aggregate. The color of the intensity profiles corresponds to the time indicated in the right color bar. (d) The front edge of the propagating fluorescent profiles, Z_{front}, is defined as the position where the fluorescence intensity reached 10% of the maximum intensity. Diffusion coefficients (D) were estimated from a linear fit of Z_{front}^2 versus t. The green and red lines show the linear fits of the data and the corresponding legends show the diffusivities estimated from the linear fits and the coefficients of determination, R^2.

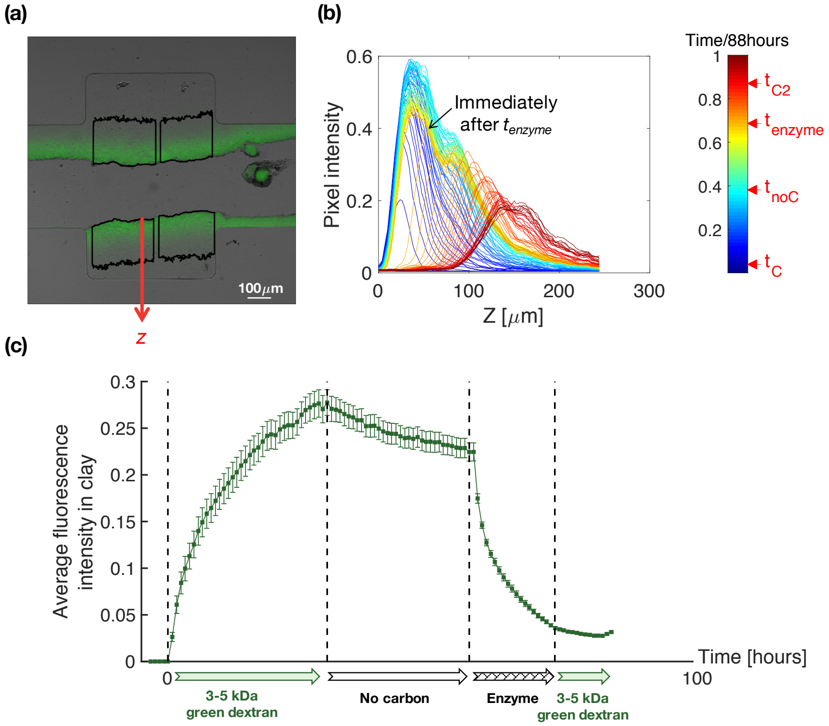

Supplementary Figure 10. No further sorption of high molecular-weight carbon to clay was observed after enzyme penetrated into clay. (a) Cross-sectional fluorescent image of a microfluidic channel containing clay micro-aggregates. (b) Fluorescence intensity profiles along the red transect, the z axis, shown in panel (a) at different times; z = 0 denotes the edge of the clay aggregate; t_C, t_{noC}, t_{enzyme}, and t_{C2} represent the start times of the injections of 3-5 kDa dextran, no organics, dextranase, and 3-5 kDa dextran (second injection), respectively. The color of the intensity profiles corresponds to the time indicated in the right color bar. (c) The symbols and error bars represent the mean and the standard error of the average fluorescence intensity within the four immobile clay regions, outlined by the four black contours in panel (a).

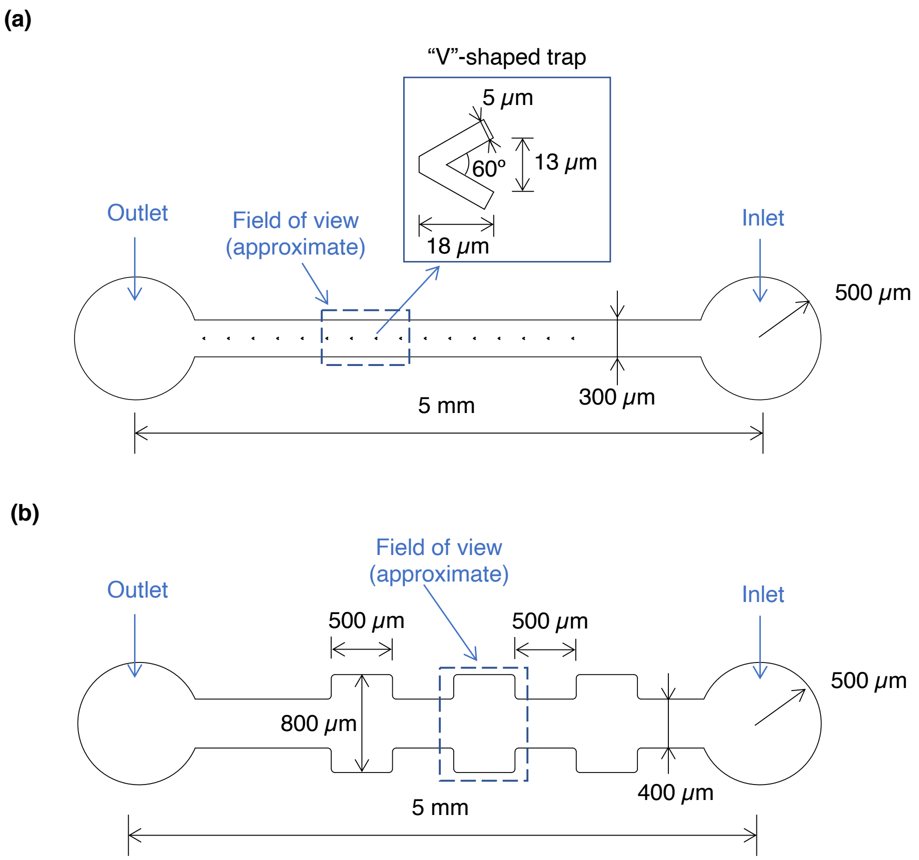

Supplementary Figure 11. The geometries of the microfluidic channels. (a) The geometry of the microfluidic channel used in Fig. 1 of the main text. The inset shows the geometry of the v-shaped clay trap in the middle of the channel. The space between the clay traps in the flow direction is 200 µm. (b) The geometry of the microfluidic channel used in Fig. 3 of the main text. The depth of both channels is 40 µm. The approximate field of the views of the confocal microscope are indicated by the blue dashed boxes.

Supplementary Discussion: Fluorescence intensity profiles of dextrans in clay further suggest a breakdown of high molecular-weight dextrans by enzyme in clay

For the dextran sorption and release experiment shown in Fig. 3, the green and red fluorescence intensity profiles along a cross-sectional transect in clay, indicated by the black z-axis in Supplementary Fig. 9(a), were tracked over time, as shown in Supplementary Fig. 9 (b) and (c), respectively. The vertical axis at z = 0 µm denotes the edge of the clay aggregate identified based on bright field images. The color of the intensity profiles indicates the time during the experiment, as shown in the right color bar. Variables t_{gC}, t_{rC}, t_{gC2}, t_{noC}, and t_{enzyme} represent the start times of the injections of 0.05 g/L green 3-5 kDa dextran, 0.05 g/L red 20 kDa dextran, 0.05 g/L green 70 kDa dextran, no organics, and 2 g/L dextranase, respectively. During the injections of green 3-5 kDa and red 20 kDa dextrans, the green and the red profiles advance into the clay, in the direction of increasing z. The front edge of the propagating fluorescent profiles, Z_{front}, is defined as the position where the fluorescence intensity reached 10% of the maximum intensity, i.e., the critical

intensity = 0.07 and 0.06 for the green and the red fluorescence, respectively. Z_{front}^2 of the green and the red fluorescence profiles at different times is shown by the green and the red crosses in Supplementary Fig. 9(d), respectively. A linear fit of Z_{front}^2 versus t during the injections of the dextrans suggests that the diffusivity of 3-5 kDa and 20 kDa dextran in clay (calculated as the slope of the linear fit divided by 4 based on the analytical solution for 1-D diffusion into a semi-infinite region from a constant concentration boundary condition, $C/C_0 = \mathrm{erfc}(z/2\sqrt{Dt})$, where erfc( ) is the complementary error function) is around 2300 $\times 10^{-12}$ cm^2/s and 40 $\times 10^{-12}$ cm^2/s, respectively. Note that as the molecular weight of the organic matter (M) increased by a factor of 4 to 7 (from 3-5 kDa to 20 kDa), the diffusion coefficient decreased by a factor of 58 (= 2300/40). This is consistent with the scaling of diffusivity for entangled polymers[2], $D \sim M^{-2.3}$ with M representing molecular weight, which suggests that for our case the diffusivity should decrease by a factor of 24 to 88.

During the injection of the enzyme dextranase, the Z_{front}^2 value of the green fluorescence front decreased, indicating a breakdown of the green 3-5 kDa dextran into lower molecular-weight molecules that desorb from the clay. In contrast, the Z_{front}^2 value of the red fluorescence front increased during enzyme injection, indicating a pronounced acceleration of the propagation of red fluorescent carbon into the clay. Consistently, the red fluorescence intensity profiles in Supplementary Fig. 9(c) show an advance of red fluorescence into the clay immediately after the enzyme injection. The diffusivity of the advancing red fluorescence during the enzyme injection, 600 $\times 10^{-12}$ cm^2/s, is over one order of magnitude larger than the diffusivity of the red 20 kDa dextran during the sorption process. This suggests that the resorbed red fluorescent carbon is much smaller than 20 kDa and thus most likely consists of fragments of red 20 kDa dextran broken down by dextranase.

To further confirm that the disappearance of fluorescence intensity in clay after enzyme injection is due to the breakdown of high molecular-weight carbon, we conducted a sorption - enzyme release - and second sorption experiment (Supplementary Fig. 10). Specifically, a buffer solution with the following substances was injected in sequence into the microfluidic soil chip shown in Supplementary Fig. 10(a): (1) 0.05 g/L green 3-5 kDa dextran, (2) no organics, (3) 2 g/L enzyme (dextranase), and (4) 0.05 g/L green 3-5 kDa dextran. During the first injection of the green dextran, the green fluorescence advanced into the clay (Supplementary Fig. 10(b)) and average fluorescence intensity in clay increased (Supplementary Fig. 10(c)), showing the sorption of the dextran to clay. Immediately after the injection of dextranase, the green fluorescence advanced further into the clay (Supplementary Fig. 10(b)), indicating a resorption of fragments of the 3-5 kDa dextran broken down by dextranase, similar to the resorption of red fluorescent carbon shown in Supplementary Fig. 9(c). In contrast with the first injection of the green dextran, the average fluorescence intensity in clay did not increase during the second injection of the dextran following the injection of the enzyme dextranase. This indicates that the exoenzyme dextranase in clay prevents the sorption of high molecular-weight carbon to clay, most likely by breaking down the high molecular-weight carbon into smaller size fragments, which then diffuse back into the solution.

Supplementary References

1. Shi, L., Zeng, M., Sun, Y. and Fu, B.M. Quantification of blood-brain barrier solute permeability and brain transport by multiphoton microscopy. *Journal of biomechanical engineering* **136**, (2014).
2. Rubinstein, M. and Colby, R.H. *Polymer Physics* **23**. (New York: Oxford university press, 2003).
