# Supplementary material for: 4D imaging reveals mechanisms of clay-carbon protection and release
Source: Nat Commun. 2021 Jan 27;12:622. doi: 10.1038/s41467-020-20798-6 (PMC7840981; doi:10.1038/s41467-020-20798-6)
Supplement: Supplementary file 7 — Description of Additional Supplementary Files [file 41467_2020_20798_MOESM7_ESM.docx]

Description of Additional Supplementary Information

Title: Supplementary Movie 1

Description: Quasi-irreversible sorption of 3-5 kDa dextran into a clay micro-aggregate.

Title: Supplementary Movie 2

Description: Reversible sorption of fluorescent glucose (340 Da) into a clay micro-aggregate.

Title: Supplementary Movie 3

Description: Desorption of dextrans with different molecular weights after the injection of enzyme dextranase (corresponds to Fig. 3). Note that near the end of the movie (after about 14 s), black dots appeared around the clay aggregates to the right of the field of view, which may be bacterial growth after the enzyme dextranase produced some glucose. Such bacterial growth should not affect our results.

Title: Supplementary Movie 4

Description: Desorption of 3-5 kDa dextrans after the injection of enzyme dextranase (corresponds to Supplementary Fig. 10).
